# PROGRESSIVE CARE OF AN AGING WOMAN: INTERACTIVE VIDEO SIMULATION CASES SPANNING 15 YEARS

**DOI:** 10.1093/geroni/igz038.3530

**Published:** 2019-11-08

**Authors:** Laurie M Kennedy-Malone, Karen Amirehsani, Rachel Zimmer, Joshua Borders

**Affiliations:** 1 School of Nursing, UNCG, Greensboro, North Carolina, United States; 2 School of Nursing, UNCG, Greensboro, United States; 3 Sticht Center for Healthy Aging and Alzheimer’s Prevention, Winston-Salem, United States; 4 School of Nursing, UNCG, North Carolina, United States

## Abstract

As a means of enhancing experiential educational opportunities for adult-gerontology nurse practitioner students who are prepared to manage the complex care of older adults, interactive simulation videos were developed using the eLearning authoring tool H5P to create learning experiences for students that can be used either in face to face classroom experiences or embedded in learning management systems. H5P is a web-based authoring tool that helps faculty build interactive course content. H5P activities provide instant feedback to students, allowing them to self-assess their understanding of the dynamic video simulation case. With funding through the Health Resources and Service Administration Advanced Nursing Education Workforce grant, four video simulation cases were developed that address emerging chronic care conditions in an older women who aged 15 years presenting initially with signs of hypothyroidism, progressed to early frailty, through moderate dementia and eventually along with her daughter face end of life health care issues. Partnering with the university instructional design experts, nurse practitioner faculty created questions that were inserted throughout the video as a means of keeping students engaged in problem-solving and decision making. A faculty handbook that described the case scenario with the interactive questions with suggested discussion questions was developed for each video simulation. The adult-gerontology primary care nurse practitioner competencies addressed in each case are identified in the handbook. Recommendations for the interactive question format will be presented and QR codes with access to direct viewing of the videos will be presented on the poster.

